# Lower Attentional Skills predict increased exploratory foraging patterns

**DOI:** 10.1038/s41598-019-46761-0

**Published:** 2019-07-29

**Authors:** Charlotte Van den Driessche, Françoise Chevrier, Axel Cleeremans, Jérôme Sackur

**Affiliations:** 1 Laboratoire de Sciences Cognitives et Psycholinguistique (LSCP), Département d’Études Cognitives de l’École Normale Supérieure, Centre National de la Recherche Scientifique, École des Hautes Études en Sciences Sociales, Paris Sciences et Lettres Research University, Paris, France; 2 Consciousness, Cognition, and Computation Group (CO3), Center for Research in Cognition and Neurosciences (CRCN), Neuroscience Institute, Université Libre de Bruxelles, Brussels, Belgium; 3 Laboratoire Interdisciplinaire de l’X, École Polytechnique, Palaiseau, France

**Keywords:** Attention, Human behaviour

## Abstract

When engaged in a search task, one needs to arbitrate between exploring and exploiting the environment to optimize the outcome. Many intrinsic, task and environmental factors are known to influence the exploration/exploitation balance. Here, in a non clinical population, we show that the level of inattention (assessed as a trait) is one such factor: children with higher scores on an ADHD (Attention Deficit/Hyperactivity Disorder) questionnaire exhibited longer transitions between consecutively retrieved items, in both a visual and a semantic search task. These more frequent exploration behaviours were associated with differential performance patterns: children with higher levels of ADHD traits performed better in semantic search, while their performance was unaffected in visual search. Our results contribute to the growing literature suggesting that ADHD should not be simply conceived as a pure deficit of attention, but also as a specific cognitive strategy that may prove beneficial in some contexts.

## Introduction

Searching for definite items in a rich environment is a fundamental behaviour in most animal species. Seemingly unrelated activities (such as foraging in the wild or looking for relevant references to include in a bibliography), all share an underlying structure: one has to navigate a predefined space so as to find items that match a definite category. Interest for this analogy is not recent in psychology and William James wrote in his *Principles of Psychology*^1^ (1890, p. 654) that “We make search in our memory for a forgotten idea just as we rummage our house for a lost object. In both cases we visit what seems to us the probable neighborhood of that which we miss. We turn over the things under which, or within which, or alongside of which, it may possibly be; and if it lies near them, it soon comes to view.” In modern terms, this suggests, on the one hand, that one cognitively represents both spatial and semantic knowledge as maps or networks^2,3^. On the other hand, the similarity between spatial foraging and internal cognitive search suggests that search processes are domain general^4^. It has been suggested that this generality could derive from the reliance on a dynamic balance of attention between exploration/exploitation that is similar in both foraging in physical space and in cognitive space^5–7^. In all domains, searches involve trade-offs between exploiting known possibilities and exploring for better opportunities elsewhere. Furthermore, there is evidence that the similarity between external and internal search processes is a consequence of evolutionary homology and not the result of convergent evolution^8^. Evidence from neuroscience, genetics and cognitive disorders substantiate the claim that molecular and neural mechanisms that evolved for the purpose of arbitrating between exploration and exploitation in the spatial domain have been recycled in later species for the control of attention^4^ (for a review see Hills *et al*., 2008). Indeed, similar dopaminergic circuits are both implicated in the regulation of goal-directed behaviour and attention, across species^4,9,10^.

Consequently, in humans, being able to regulate one’s attention is closely related to behavioral control^11^. A deficit of this ability is the core symptom of Attention Deficit/Hyperactivity disorder (ADHD)^12,13^, a mental disorder characterized by a reduced ability to focus and sustain attention, and by an excessive level of activity. Dopamine deficit is currently the leading theory for explaining ADHD^14^, as notably, behaviours associated with ADHD have been correlated with specific polymorphisms alleles coding for dopaminergic proteins^15–17^. Individuals diagnosed with ADHD also show elevated levels of the dopamine transporter, responsible for moving dopamine out of the synaptic cleft^18^. Lower levels of dopamine in the synapse, arguably contributes to the inability to focus, as well as to behaviours that appear to be related to novelty-seeking^19,20^. In addition, the reference drug for treating ADHD, methylphenidate, increases synaptically released dopamine^17,21^. ADHD and especially the hyperactive subtype is also associated with extreme novelty seeking^22^, and genes implicated in the dopaminergic pathways, associated with ADHD^23^ (DRD4 allele variants, see Hawi *et al*., 2003 for a review) are more frequent in populations that have a history of migration^24^. Together, this evidence has led to the notion that ADHD might be part of an adaptation to a threatening and food scarce environment our ancestors lived in^25^. In the present environment, studies have shown a correlation between the number of regions visited when free-viewing a visual scene with curiosity as a personality trait^26^ and also ADHD^27^. Together, this suggests that attention deficits in ADHD would co-vary with a bias towards exploration in the regulation of the exploration/exploitation trade-off, and explain some behavioural patterns of activity found in ADHD.

**Figure 1:**
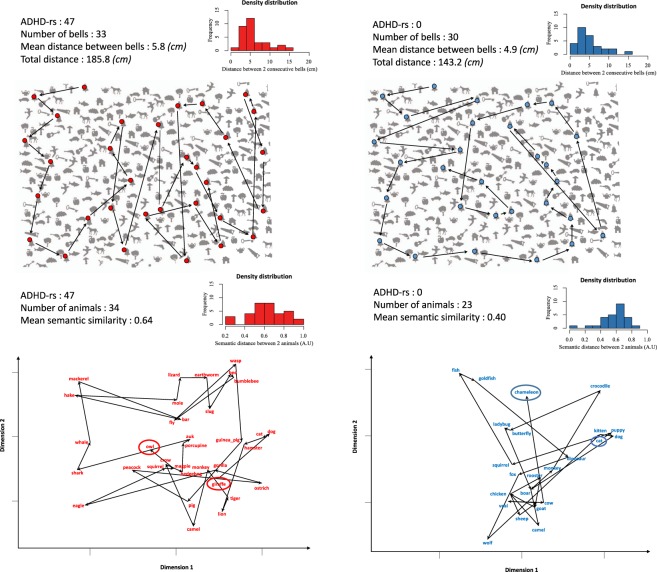
Illustration of search paths in visual (top, with the stimulus sheet overlaid – Bells test by Gauthier *et al*. 1989^30^) and semantic (bottom) spaces for two participants, representative of diverse patterns of Inattentive/hyperactive behaviours, one with high (47, left, red) and the other with low (0, right, blue) rating on the ADHD-rating scale. For illustrative purposes only, we here represent the semantic space by means of the *t*-distributed stochastic neighbor embedding (*t*-SNE, *tsne* package in *R*^56^) algorithm for dimensionality reduction, in order to obtain a two dimensions map of the 300 dimensions vectors from the word2vec google.news model^34^. Insets contain the histograms for the distributions of distances.

In this paper we ask whether this preference for exploration over exploitation with increasing attention deficit traits is found in a non-clinical population. Indeed, ADHD symptomatology can be viewed as a continuum^28^, as most of the traits are found at a variable degree in the general population. Thus we predicted that children with lower abilities to sustain attention would adopt search strategies that favour exploration over exploitation in search tasks. Following Hills *et al*. (2008)^4^, we further inquire whether this preference would generalize across external and internal search domains, and to that end, we relied on two classical neurospychological search tasks tapping selectively into each domain. We tested a population of 87 neurotypical children whose general behaviour and attention profile were evaluated on the ADHD rating scale^29^. For the external search we used the bells test^30^, where participants are asked to find as many silhouette drawings of bells among a set of distractors, in a limited space - an A4 paper sheet - and time - 2 minutes. For the internal search we used a semantic fluency task, where participants are required to search in memory for words corresponding to a semantic category (animals) and name them aloud. We analyzed both tasks as foraging in visual and semantic spaces^7^. Recently, measures of semantic similarity by means of word-embedding methods have been tested in semantic verbal fluency^31,32^. They stand out as one the most objective methods to measure a semantic distance between productions in semantic fluency tasks, and comparable to euclidean distance in visual search. We predicted that children with higher scores on the ADHD-rs should produce more long distance ‘jumps’ between consecutive items, as a consequence of more explorative traits (see Fig. 1).

## Materials and Methods

### Participants

Participants were 87 children (8 to 11 years old, mean age 9,3 (sd = 0.93), 36 girls/51 boys, all in fourth grade) from 4 different schools in Saint-Malo, Britany, France. All children were native French speakers. For each child, parents and teachers in their respective classes filled the ADHD-r^33^, in order to obtain a measure of their Attentive/Hyperactive behaviours. The ratings ranged from 0 to 50 (the maximum score on the scale is 54), with a mean ADHD-rs score of 13.8 (SD = 13.33) and a median of 9. ADHD-rs evaluate 18 behaviours (9 items related to inattention and 9 related to hyperactivity/impulsivity). Note that the ADHD-rs is here used only as a means to evaluate the cognitive dimension that is at the core of the disorder. It has no clinical intent or application. The research was approved by the local ethics committee (comité de protection des personnes d’Ile de France) and conducted according to the Declaration of Helsinki. Informed consent was obtained from parents and teachers of all participants.

### Design, protocol and analysis

Children performed individually two tasks: a visual and a semantic search task in one session. The order of assessment was random. They were informed that they would perform two exercises concerning their attention. In the analyses, we used the teacher’s ADHD scores, on the basis that they had a better overall frame of reference, yet we used parents’ scores as a control and excluded 3 children for whom the parents’ scores was greater than teachers scores by more than one standard deviation.

#### Visuo-spatial search task

We used the Bells Test^30^. The test stimulus was an A4 paper sheet containing black on white silhouettes of common objects (see Appendix 1), with bells designed as targets. 35 targets were distributed equally in 7 columns, each column having the same number of targets (N = 5) and distractors (N = 40) with a balanced number of targets in each quadrants. Children were instructed to cross-out as many bells as they could in a two minutes interval. While participants performed the task, an experimenter registered on the scoring sheet the order of the bells found. Participants started from a bell in the upper left corner (Fig. 1).

#### Semantic search task

Participant had to retrieve from their memory and name aloud as many words from the category “animal” as they could in two minutes. Performance was recorded, transcribed and time-stamped off-line.In order to use word embedding with pre-trained vectors on part of Google News dataset^34^, we translated children’s productions from French into English. Word-embedding is a method of language modeling and feature learning techniques in natural language processing (NLP), where words or phrases from the vocabulary are mapped to vectors of real numbers. This method involves a mathematical embedding from a space with one dimension per word to a continuous vector space and has been demonstrated to be a relevant method to measure semantic distance between words^35^. We performed the analysis using the Python implementation of word2vec in the Gensim package^36^. This method gives a vectorial representation of words in 300 dimensions, and the cosine of two vectors provides a measure of the semantic similarity of two words, ranging from 0 (no similarity) to 1 (identity, see Mikolov *et al*.^35^). We based our analyses on (1 - similarity) to obtain a measure of distance, as with visual search (see Fig. 1).

We ran generalized mixed models with performance in the search tasks as dependant variables and ADHD score as independent variable and subject as random factor. To statistically test differences in long distance of distributions we used two methods: First, we performed mixed effect quantile regressions from quantile 0.5 to 0.95 by steps of 0.05, on the distributions of raw distances, with the ADHD score as predictor and participants as grouping factor. Second, we splitted participants in highs and lows with respect to the median (m = 9) of the population scores, and for each quantile, from 0.5 to 0.99 in steps of 0.01, of the global distribution of distances, we applied a Poisson regression on the number of distances above said quantile for each child, with group (high or low ADHD score) as a predictor. We tested significance by means of bootstrapping (N = 100) with a cluster-based significance level of 0.05. We performed statistical analysis using *R* (R Core Team, 2014), with the *lme4* package for mixed models^37^, the *lmerTest* package to perform likelihood ratio tests^38^ and the *lqmm* package for quantile regressions^39^. Raw data, translation of the lists of words and analyses scripts are available on the Open Science Framework at https://osf.io/2n4q9/.

## Results

### Visual search

**Figure 2:**
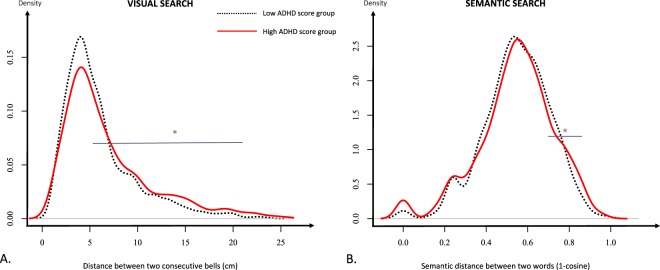
Distribution of distances between two consecutive targets in the visual search task (**A**) and in the semantic search task (**B**), for children scoring high (pain lines) and low (dotted lines) on the ADHD rating scale (ADHD-rs), according to a median split (m = 9) on the scores. The horizontal bars correspond to the range of a significant difference in the density distributions, assessed by means of bootstrap with a cluster-wise significance level of 0.05–from 0.56 to 0.99, for the visual search task and from 0.87 to 0.99 for the semantic search task. On the semantic search task, the distribution of similarities shows two minor peaks: the first one at 0 corresponds to the immediate repetitions (ex: dog-dog). The second one correspond to the “dog-cat” pairs with semantic similarity of 0.29.

Children found a mean of 30.1 (*SD* = 3.0) bells within two minutes. No difference were found depending on ADHD score (p > 0.7). We computed the distances between consecutive bells in each participant’s search path. Search paths of children with higher ADHD ratings were more variable and contained more long distances: we found a positive and significant effect of the ADHD score on both the mean (β = 0.026, *SD* = 0.0096, *z* = 2.71, *p* < 0.01) and the standard deviation (β = 0.035, *SD* = 0.008, *z* = 4.36, *p* < 0.0001) of distances. Inspection of the distribution of these distances (Fig. 2A) suggests that these effects are due to an excess of long transitions in participants with higher scores, as the distribution is positively skewed, and the more so for high ADHD ratings participants. We statistically tested this observation by means of two methods: First, we performed mixed effect quantile regressions from quantile 0.5 to 0.95 by steps of 0.05, on the distributions of raw distances, with the ADHD score as predictor and participants as grouping factor. We found a positive effect of the ADHD score (all *ps* < 0.05) for the quantiles 0.6, 0.8, 0.9 and 0.95 (see Supplementary Table 1). Second, we splitted participants in highs and lows with respect to the median (m = 9) of the population scores, and for each quantile, from 0.5 to 0.99 in steps of 0.01, of the global distribution of distances, we applied a Poisson regression on the number of distances above said quantile for each child, with group (high or low ADHD score) as a predictor. By means of bootstrapping (N = 100), with a cluster-based significance level of 0.05, we found that the two groups differed from the quantile 0.56 to the quantile 0.99 (see Fig. 2A), with participants high on the ADHD-rs having, again, an excess of long distances compared to participants low on the scale.

Thus, children with higher ratings on the ADHD scale “travelled” on average significantly longer distances between two bells, notably because they inserted more “long jumps” in their search paths, but without incurring significant costs to their performance.

### Semantic search

Children named on average 26.9 (*SD* = 8.1) animals within two minutes. Scores on the ADHD-rs were positively correlated with higher production (total number of items–β = 0.005, *SD* = 0.001, *z* = 3.477, *p* < 0.001, Poisson regression for count data), but also with higher performance (number of different animals–β = 0.004, *SD* = 0.0016, *z* = 2.49, *p* < 0.02), as previously reported within the non-clinical population^8^. They also repeated themselves more often: in a Poisson regression on the count of immediate repetitions with ADHD score and performance as predictors, we found significant and positive effects of both the ADHD score (β = 0.017, *SD* = 0.007, *z* = 2.33, *p* = 0.02, see Fig. 2B) and performance (β = 0.049, *SD* = 0.012, *z* = 4.006, *p* < 0.001), meaning that the increased number of repetitions is present even while controlling for number of items produced. There was no effect of ADHD ratings on distant repetitions (*p* > 0.2).

Next, as a test of the relation between the corpus-based semantic distances and children’s behaviour, we asked whether Inter item Response Time (IRT) and semantic distances were correlated. We found (see Supplementary Fig. S1) that more distant animals in semantic space yielded longer IRTs (β = 8.203, *SD* = 0.675, df = 2177, *t* = 12.147, *p* < 0.0001–mixed effect linear regression with participants as random factor). This is coherent with the notion that when searching for animals, children behave as explorers, so that more distant positions in semantic space yield longer travel times.

Next, for each child we computed the mean of the distances in her/his search path, as well as the standard deviation. By means of linear regressions with the ADHD score as predictor, we found that the mean distance did not depend on ADHD traits (*p* > 0.3). However, we found a significant and a positive effect on the standard deviation (β = 0.0006, *SD* = 0.0003, *z* = 2.38, *p* < 0.02) of the distances, meaning that the variability of the search paths increased with increasing ADHD ratings. Inspection of Fig. 2B suggests that this difference is in part due to the inclusion of more long distance semantic jumps in the search paths of participants with higher ADHD ratings. We statistically tested this observation with the same two strategies as for the visual search task: First, we performed mixed effect quantile regressions from quantile 0.5 to 0.95 by steps of 0.05, on the distributions of raw distances, with ADHD score as predictor and participants as grouping factor. We found a positive main effect of the ADHD score for the 0.95 quantile (β = 0.012, *SD* = 0.006, IC-95 = [0.001; 0.02], *p* = 0.03–see Supplementary Table 2). Second, with the same median split as above, for each quantile, from 0.5 to 0.99 in steps of 0.01 of the global distribution of semantic distances, we applied a Poisson regression on the number of distances above said quantile for each child, with group as a predictor. By means of bootstrapping (N = 100), with a cluster-based significance level of 0.05, we found that the two groups differed from the quantile 0.87 to the quantile 0.99 (see Fig. 2B), with participants high on the ADHD-rs having, again, an excess of long distances compared to participants low on the scale.

Thus, children with higher ratings on the ADHD scale produced more long distances in semantic search, but here this was accompanied by an increase in performance.

Finally we tested whether behaviours in visual and semantic searches were correlated; we computed an index for visual and semantic search by summing for each subjects the quantile that are significant according to the quantile regression (i.e. for the quantiles 0.60, 0.80, 0.90 and 0.95 to the visual search and the quantile 0.95 for the semantic search). The linear regression between the two indices controlled for the ADHD score was not significant (p > 0.3).

## Discussion

In this study we found that for two simple search tasks, one external and internal, children who scored higher on the ADHD-rs produced more long distances and were also more variable than those who scored lower on the ADHD-rs. These results are in line with the literature on variability in ADHD^40^. Importantly, we found these effects in the tails of the distributions and not at their peaks, suggesting that the differences between children stem from more frequent long jumps with higher ADHD score, rather than from a global search process modification: while all children use a similar base local search strategy, children with higher scores on the ADHD-rs inserted more often jumps to distant positions in the search space. Thus, their tuning of the exploration/exploitation ratio is biased in favor of exploration, without decrement in performance. Our results are also coherent with previous studies on each search task. Performance in visual search is lower in children with ADHD^41,42^. In semantic search, the number of words cited is higher in participants with ADHD^43,44^. Yet, interestingly, when the fluency is phonological their performance is lower^45,46^ which agrees with the notion that phonological search requires more executive control than semantic search^47^. This difference of performance in different searches might comes from the nature of the environment. Indeed, in our visual search task, targets were uniformly distributed, while targets in our semantic search are plausibly more sparsely and unevenly distributed. We speculate that this distribution could have a greater homology with distributions of targets in natural foraging environments.

Along this line, we would suggest that the bias in favor of exploration is a latent cognitive trait that is diversely expressed depending on the task context. Thus, the same exploration bias may have differential impacts on performances depending on search domains, and could even become beneficial as in our semantic task. This is coherent with the evolutionary interpretation of ADHD according to which impulsive and unstable behaviours while maladaptive in contemporary contexts, may have been more often beneficial in ancestral contexts^25^. The diversity of natural search environments might have promoted the emergence of a diverse set of search strategies, contributing to the relatively high prevalence of ADHD traits^48^. Following these lines, our results contribute to the mounting evidence that lower inhibition in ADHD might enhance creativity^49,50^, as a result of more exploratory behaviours. Here we should note that, in the visual search task, all children explored the same visual stimulus while the semantic space that was the base of the semantic search was idiosyncratic. Further research is needed to investigate the role of this distinction in potentiating the benefit of more exploratory search strategies. It is of prime importance to note, however, that our children participants were all healthy children, and that those who scored high on the ADHD-scale were not clinical ADHD. Thus, in clinical populations, some other clinical dimensions of ADHD such as motivation or vigilance disorder^51–53^ might come into play and mitigate the benefits of the exploratory strategy found in our results.

Though we did not directly measure dopamine, our results provide insight for the proposal that search processes and regulation of attention are goal directed behaviours that share a general cognitive basis that depends on dopaminergic pathways^4,8^. The group level consistency of these exploring/inattentive phenotypes suggests some corresponding genotypes especially for genes coding for dopaminergic systems. Further studies might explore the link between exploring and inattentive behaviours and the variability in the genes coding for the dopaminergic system. In addition it might be of interest to take into account the intra-individual variability of behavior, as it is a consistent feature of ADHD and also represents a crucial point in natural selection by determining the behaviour of prey and predators^54^.

Turning now to the subjective component of ADHD, we suggest that jumps in searches could be the overt manifestations of attentional lapses. Indeed, we propose that blank thoughts, during which no mental content can be reported and that we previously identified as more frequent in ADHD^55^, might be caused by periods of rapid shifts in mental contents, also named “transitive states” by James^1^, during which introspection is difficult. Blanks would thus not be true episodes of empty thoughts, but by-products of explorative attentional strategies in mental space. Further research is thus needed to investigate this hypothetical functional role of mental states subjectively experienced as blanks.

## Supplementary information

appendix and supplementary materials

## Data Availability

The datasets generated and analysed during the current study are publicly available via the Open Science Framework and can be accessed at https://osf.io/2n4q9/.
