# Supplementary material for: Lower Attentional Skills predict increased exploratory foraging patterns
Source: Sci Rep. 2019 Jul 29;9:10948. doi: 10.1038/s41598-019-46761-0 (PMC6662844; doi:10.1038/s41598-019-46761-0)
Supplement: Supplementary file 1 — appendix and supplementary materials [file 41598_2019_46761_MOESM1_ESM.pdf]

Lower Attentional Skills predict increased exploratory foraging patterns

Charlotte Van den Driessche[1,2*], Françoise Chevrier[1], Axel Cleeremans [2], Jérôme Sackur[1,3]

1. Laboratoire de Sciences Cognitives et Psycholinguistique (LSCP), Département d'Études Cognitives de l'École Normale Supérieure, Centre National de la Recherche Scientifique, École des Hautes Études en Sciences Sociales, Paris Sciences et Lettres Research University;
2. Consciousness, Cognition, and Computation Group (CO3), Center for Research in Cognition and Neurosciences (CRCN), Neuroscience Institute, Université Libre de Bruxelles;
3. Laboratoire Interdisciplinaire de l'X, École Polytechnique

* Corresponding authors: Charlotte Van den Driessche charlotte.vandend@gmail.com
Permanent Address: Laboratoire de Sciences Cognitives & Psycholinguistique,
École Normale Supérieure, 29 rue d'Ulm, 75005, Paris, France
Tel: +33 1 44 32 26 25
Center for Research in Cognition and Neurosciences (CRCN),
Université Libre de Bruxelles (ULB),
Avenue F.D. Roosevelt 50, CP191, 1050 Brussels, Belgium
Tel: +32 2 650 26 33

Appendix

Bells Test task sheet presented to the subject (Gauthier, L., Dehaut, F., & Joanette, Y., 1989)

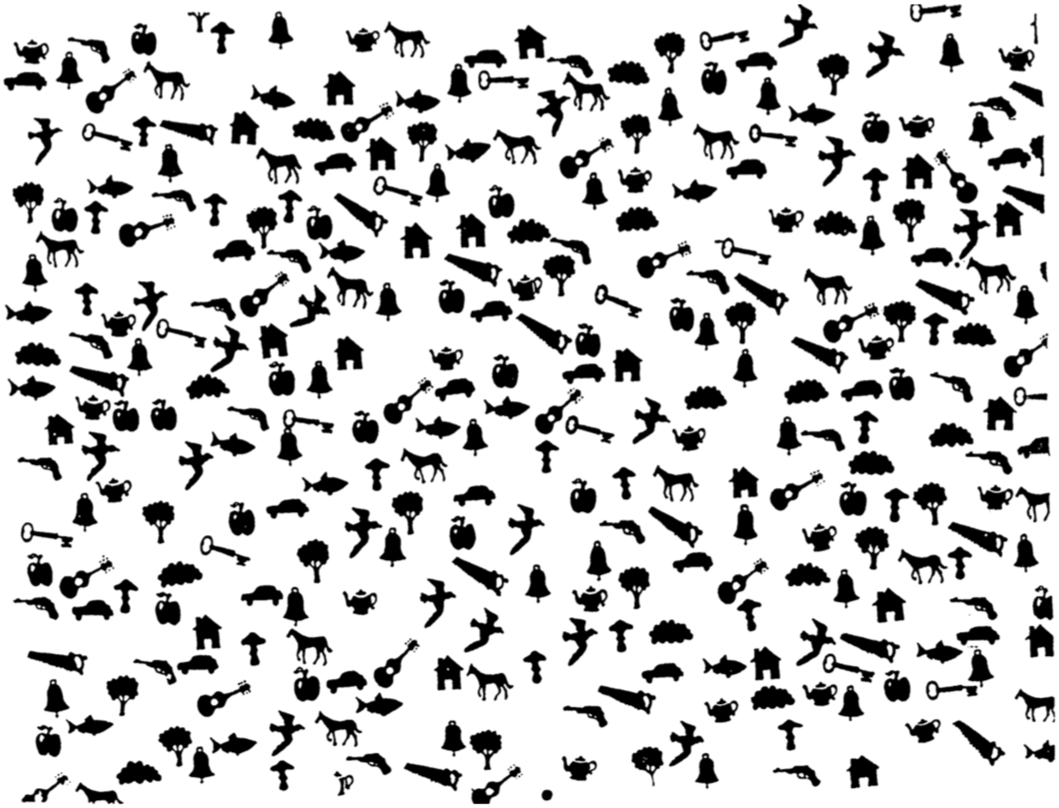

SUPPLEMENTARY MATERIAL

Quantile	β	*SD*	Lower bound	Upper bound	*p*-value	
0.5	0.017336	0.114375	-0.212509	0.2472	0.8801	n.s
0.55	0.16121	0.14210	-0.12435	0.4468	0.2621	n.s
0.6	0.346589	0.150686	0.043774	0.6494	0.02574	*
0.65	0.375577	0.201541	-0.029435	0.7806	0.06839	.
0.70	0.44499	0.28626	-0.13028	1.0203	0.1265	n.s
0.75	0.44540	0.33672	-0.2312	1.1221	0.192	n.s
0.80	0.61899	0.25804	0.10045	1.1375	0.0203	*
0.85	0.792445	0.417480	-0.046513	1.6314	0.06357	.
0.90	1.01396	0.45054	0.10856	1.9194	0.02894	*
0.95	1.32269	0.48233	0.35342	2.292	0.008495	**

Table 1 .main effect of the ADHD score on the distribution of distances between two retrieved bells from quantile .5 to .95.

Quantile	β	*SD*	Lower bound	Upper bound	*p*-value	
0.5	-0.0003880	0.0042708	-0.0089705	0.0082	0.9280	n.s
0.55	0.00073578	0.00711662	-0.01356561	0.0150	0.9181	n.s
0.6	0.0019960	0.0048056	-0.0076612	0.0117	0.6797	n.s
0.65	-0.00012394	0.01545858	-0.03118911	0.0309	0.9936	n.s
0.70	0.0030335	0.0311530	-0.0595708	0.0656	0.9228	n.s
0.75	0.00010246	0.00541670	-0.01078281	0.0110	0.9850	n.s
0.80	2.9396e-03	7.2145e-03	-1.1558e-02	0.0174	0.6854	n.s
0.85	0.0059539	0.0064899	-0.0070880	0.0190	0.3634	n.s
0.90	0.0070891	0.0066748	-0.0063243	0.0205	0.2934	n.s
0.95	0.01240040	0.00574337	0.00085867	0.0239	0.03577	*

Table 2 .main effect of the ADHD score on the distribution of distances between two cited animals from quantile .5 to .95.

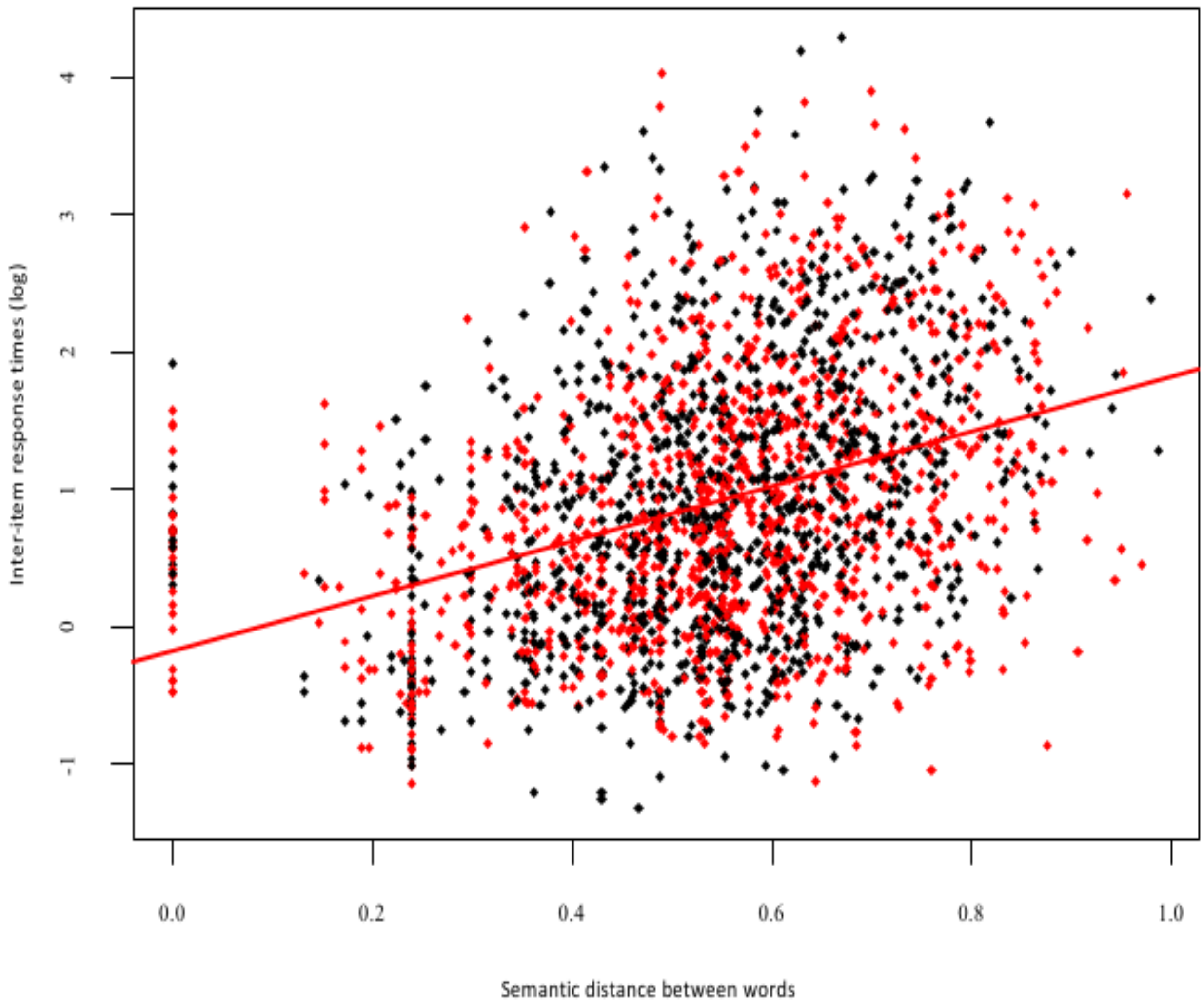

<u>Fig. S1</u> Semantic distance of all animal pairs produced versus Inter-item Response Time. In red the pairs produced by participants with a higher score on ADHD-rs and in black participants with a low score on ADHD-rs.

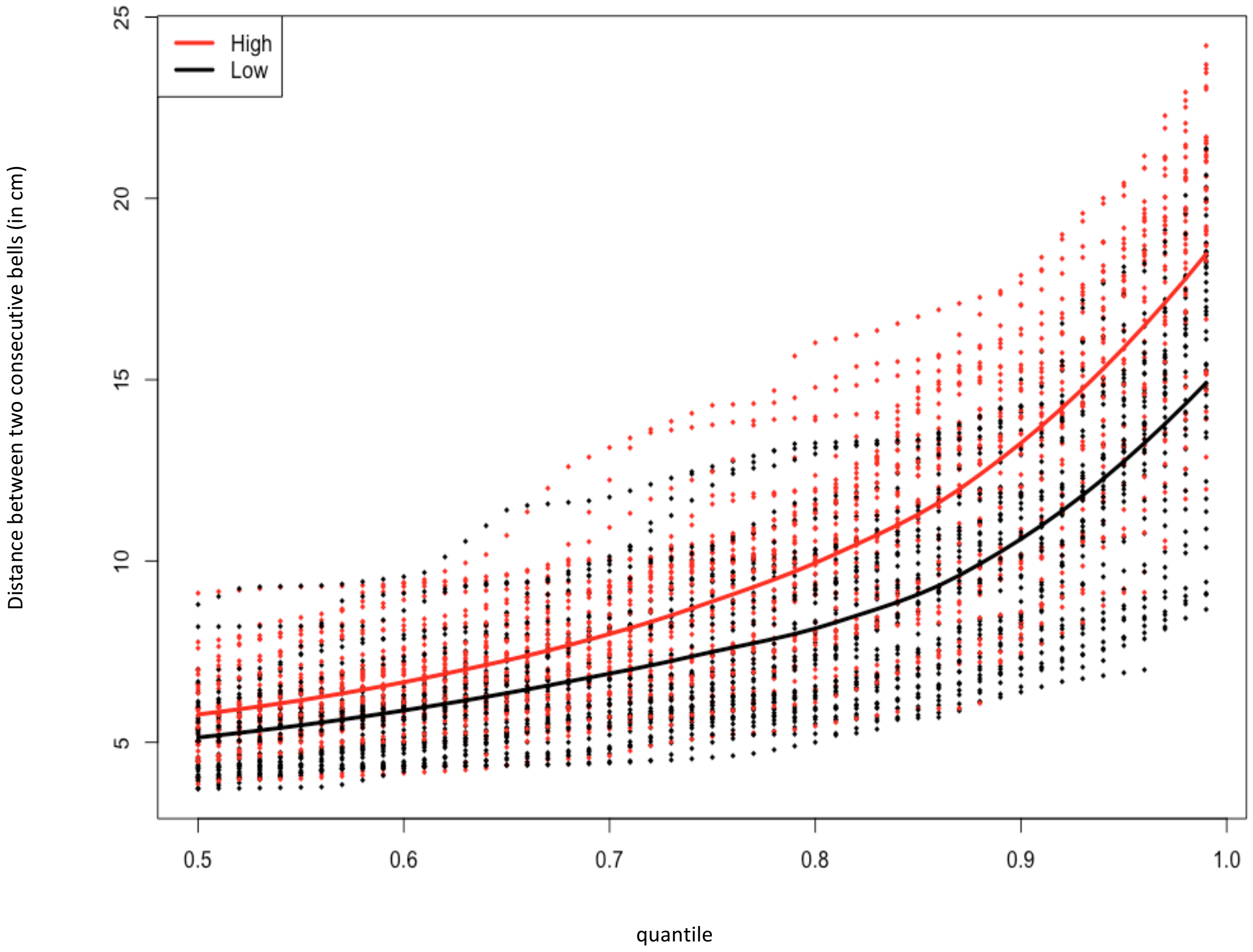

FIG.S2A *Plot of the 0.5 to 0.95th quantile of each participant. In red the high group and in black the low group showing that values of the quantiles are higher for participant showing a high level of adhd behaviours.*

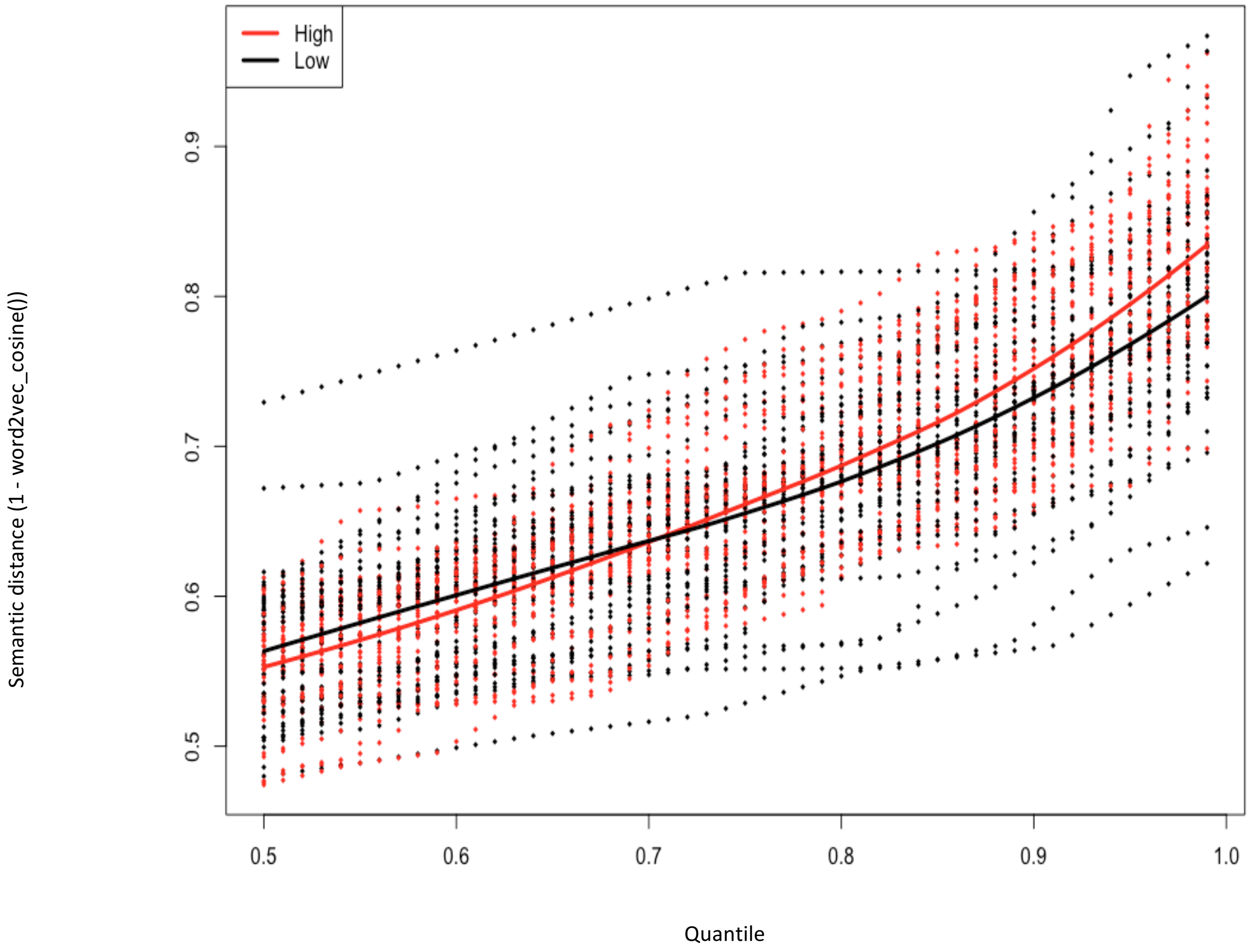

FIG.S2B *Plot of the 0.5 to 0.95th quantile of each participant. In red the high group and in green the low group showing that values of the quantiles are lower for participant showing a high level of adhd behaviours.*
